# Smoke Condensate-Induced Vascular Senescence and SASP Are Attenuated by Dual mTORC1/2 Inhibition with Rapalink-1

**DOI:** 10.3390/ijms27083636

**Published:** 2026-04-19

**Authors:** Jinliang You, Hongjun Liu, Dilaware Khan, Robert Muhereza, Katharina Faust, Sajjad Muhammad

**Affiliations:** Department of Neurosurgery, Medical Faculty, University Hospital Düsseldorf, Heinrich-Heine-Universität Düsseldorf, Moorenstr. 5, 40225 Düsseldorf, Germany; jinliangyou8@gmail.com (J.Y.); hongjunliu303@gmail.com (H.L.); dilaware00@yahoo.com (D.K.); robertlana0@gmail.com (R.M.); katharinaangela.faust@med.uni-duesseldorf.de (K.F.)

**Keywords:** rapalink-1, mTOR, vascular senescence, SASP

## Abstract

Cigarette smoking contributes to vascular aging through oxidative stress, inflammation, and extracellular matrix (ECM) remodeling. Cellular senescence has been recognized as an important mechanism linking tobacco exposure to vascular dysfunction, but effective pharmacological strategies targeting this process remain scarce. In this study, we examined whether Rapalink-1, a dual inhibitor of mechanistic target of rapamycin complex 1 and complex 2 (mTORC1 and mTORC2), modulates smoke condensate (SC)-induced senescence in vascular cells. Human umbilical vein endothelial cells (HUVECs) and vascular smooth muscle cells (SMCs) were exposed to SC with or without Rapalink-1. SC increased intracellular reactive oxygen species, induced DNA damage, and promoted senescence-associated changes, including increased senescence-associated β-galactosidase (SA-β-gal) activity, reduced Lamin B1, and elevated p21 expression. These effects were accompanied by increased expression of inflammatory and matrix-remodeling genes associated with the senescence-associated secretory phenotype (SASP). Rapalink-1 co-treatment reduced oxidative stress and DNA damage, attenuated senescence markers, and partially normalized SASP-related and ECM-associated gene expression. Mechanistically, SC activated nuclear factor kappa B (NF-κB) and mitogen-activated protein kinase (MAPK) signaling and increased downstream mTOR pathway activity, whereas Rapalink-1 dampened these signaling responses. Together, these findings indicate that dual mTORC1/2 inhibition by Rapalink-1 mitigates smoke condensate-induced senescence and inflammatory responses in vascular cells.

## 1. Introduction

Tobacco smoking remains one of the leading preventable causes of cardiovascular and cerebrovascular morbidity and mortality worldwide, strongly predisposing individuals to atherosclerosis, intracranial aneurysms, and stroke [1,2,3,4]. Beyond its systemic toxicity, cigarette smoke exerts profound deleterious effects on the vascular wall by promoting oxidative stress, chronic inflammation, and extracellular matrix (ECM) remodeling, thereby accelerating vascular aging and structural degeneration [5,6,7,8]. Epidemiological evidence consistently demonstrates that smokers exhibit an increased incidence of vascular disease and worse clinical outcomes compared with non-smokers [9,10,11]. However, the intracellular signaling networks that mechanistically link tobacco exposure to vascular degeneration remain incompletely understood.

Accumulating evidence suggests that cellular senescence is an important mechanism linking tobacco smoke exposure to vascular aging [12,13]. Persistent oxidative and genotoxic stress induced by cigarette smoke drives premature senescence in both endothelial cells and vascular smooth muscle cells. Senescent vascular cells display characteristic molecular features, including increased senescence-associated β-galactosidase (SA-β-gal) activity, depletion of nuclear Lamin B1, accumulation of DNA damage markers such as phosphorylated H2A histone family member X (γ-H2AX) and 8-hydroxy-2′-deoxyguanosine (8-OHDG), and sustained cell-cycle arrest [14,15,16]. Importantly, senescent cells acquire a senescence-associated secretory phenotype (SASP), marked by excessive production of pro-inflammatory cytokines, chemokines, and matrix-degrading enzymes, which collectively amplify local inflammation and ECM disruption. Through these processes, cellular senescence contributes to vascular wall weakening, maladaptive remodeling, and disease progression in conditions such as aneurysm formation, atherogenesis, and vascular stiffening [17,18,19,20]. Despite its pathogenic relevance, effective pharmacological strategies to suppress smoke-induced vascular senescence remain limited.

To dissect these mechanisms under controlled conditions, tobacco smoke condensate (SC)—the particulate phase of cigarette smoke—is widely employed as an in vitro model of smoke-induced vascular injury [21,22,23]. SC exposure robustly induces reactive oxygen species (ROS) accumulation, mitochondrial dysfunction, and DNA damage in endothelial and smooth muscle cells, ultimately triggering premature senescence [12,22,24]. These stress responses converge on key signaling pathways, including nuclear factor kappa B (NF-κB) and mitogen-activated protein kinase (MAPK), which orchestrate inflammatory and stress-adaptive transcriptional programs, as well as the mTOR pathway, a central regulator of cellular metabolism, growth, and aging. Persistent activation of these pathways reinforces oxidative and inflammatory signaling, promotes SASP activation, and drives maladaptive ECM remodeling—hallmarks of smoking-associated vascular degeneration. However, therapeutic approaches targeting individual downstream consequences, such as antioxidants or anti-inflammatory agents, have shown limited efficacy, underscoring the need for interventions that modulate upstream signaling nodes governing senescence and stress integration.

The mechanistic target of rapamycin (mTOR) pathway has emerged as a master regulator linking nutrient sensing, stress responses, and cellular aging. Chronic mTOR activation accelerates oxidative stress, inflammatory signaling, and senescence, whereas mTOR inhibition extends lifespan and alleviates age-related pathologies across multiple species [25,26,27,28]. Rapalink-1 is a third-generation dual mTORC1/2 inhibitor that overcomes limitations of earlier mTOR inhibitors and exhibits enhanced potency and durability [29,30]. Beyond its established roles in cancer and metabolic disease, recent studies demonstrate that Rapalink-1 confers cytoprotective effects by coordinately suppressing NF-κB, MAPK, and mTOR signaling, thereby attenuating stress-induced cellular dysfunction [31]. Notably, dual mTORC1/2 inhibition also impacts protein kinase B (AKT) signaling, a critical stress-adaptive pathway implicated in cellular survival and homeostasis, suggesting that Rapalink-1 may fine-tune signaling networks rather than simply abrogate them.

Based on these considerations, we hypothesized that Rapalink-1 protects vascular endothelial and smooth muscle cells from tobacco smoke condensate-induced oxidative stress, DNA damage, and premature senescence by reprogramming interconnected inflammatory and metabolic signaling pathways. By integrating cellular, molecular, and transcriptional analyses, the present study aimed to determine whether dual mTORC1/2 inhibition by Rapalink-1 mitigates SC-induced vascular injury through coordinated modulation of NF-κB/MAPK signaling, mTOR signaling to ribosomal protein S6 (S6) output, and stress-adaptive AKT activation. This work provides mechanistic insight into how targeting senescence-associated signaling networks may offer a promising therapeutic strategy to counteract smoking-related vascular aging and degeneration.

## 2. Results

### 2.1. Rapalink-1 Alleviates SC-Induced Oxidative Stress and Restores Cell Viability in HUVECs and SMCs

To determine whether SC induces oxidative stress and whether Rapalink-1 can counteract this response, intracellular ROS levels were first evaluated using 2′,7′-dichlorodihydrofluorescein diacetate (DCFH-DA) staining.

**Figure 1:**
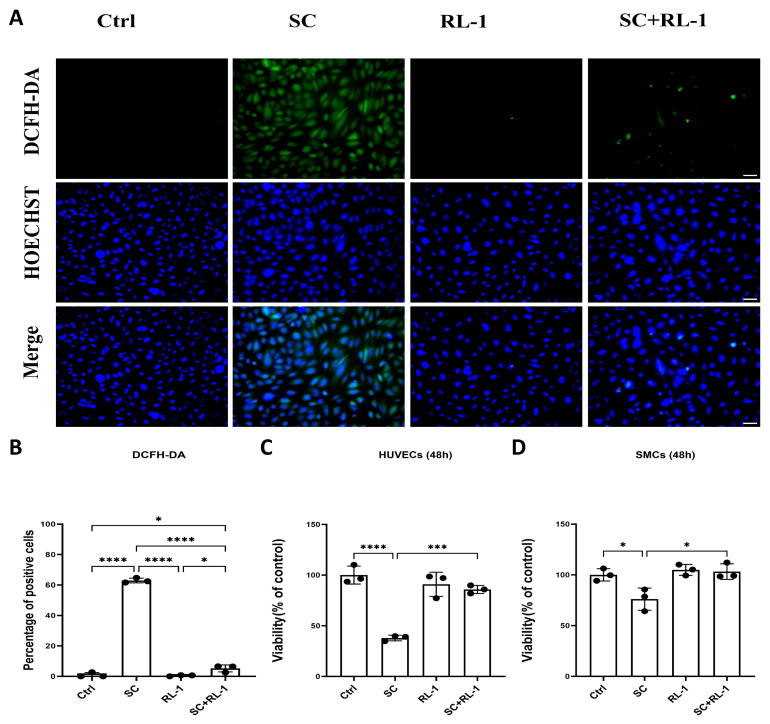
(**A**) Representative fluorescence images of DCFH-DA staining (green) with Hoechst nuclear counterstaining (blue) in HUVECs after 2 h treatment under four conditions: Control (Ctrl), SC, Rapalink-1 (RL-1), and SC + RL-1. (**B**) Quantification of DCFH-positive cells (%) in HUVECs. (**C**,**D**) Cell metabolic viability measured by MTT assay in HUVECs and SMCs at 48 h. Scale bar = 50 μm, Data are presented as mean ± SD (*n* = 3). Statistical significance was determined by one-way ANOVA with Tukey’s multiple comparisons test. Significance is indicated as: * *p* < 0.05, *** *p* < 0.001, **** *p* < 0.0001.

In HUVECs, SC markedly increased DCFH-DA-derived fluorescent signals, indicating elevated intracellular oxidative stress, whereas Rapalink-1 alone did not appreciably affect basal staining. Notably, co-treatment with Rapalink-1 significantly reduced the SC-induced increase in DCFH-DA-positive cells (Percentage of positive cells: Control = 0.90 ± 1.56%; SC = 62.93 ± 1.58%; Rapalink-1 = 0.49 ± 0.43%; SC + Rapalink-1 = 5.20 ± 2.30%; *p* < 0.0001; Figure 1A,B). In SMCs, a similar oxidative response was observed following SC exposure (Supplementary Figure S1).

Consistent with these findings, SC stimulation induced a coordinated oxidative stress-related transcriptional response in both HUVECs and SMCs, including NOX4 and NOS2, as well as activation of antioxidant defense pathways such as HMOX1, SOD2, and the redox-responsive transcription factor NFE2L2. Rapalink-1 alone also caused modest changes in a subset of transcripts in HUVECs, although these were generally smaller than the SC-induced responses. Importantly, Rapalink-1 co-treatment selectively attenuated parts of this stress-responsive gene program, with the strongest suppression observed for NOS2, SOD2, and NFE2L2, whereas in HUVECs, HMOX1 remained elevated despite Rapalink-1 treatment (Table 1 and Table 2).

We next examined whether the reduction in oxidative stress translated into improved cellular function. In HUVECs, SC significantly impaired metabolic viability at 48 h, whereas Rapalink-1 alone or in combination with SC preserved viability. Similarly, in SMCs, SC exposure reduced cellular viability, while Rapalink-1 co-treatment restored viability to near-control levels (Figure 1C,D).

### 2.2. Rapalink-1 Attenuates SC-Induced DNA Damage and Oxidative DNA Lesions in Vascular Cells

Because oxidative stress and genotoxic injury are closely linked, we next evaluated DNA damage using γ-H2AX and 8-OHDG staining.

**Figure 2:**
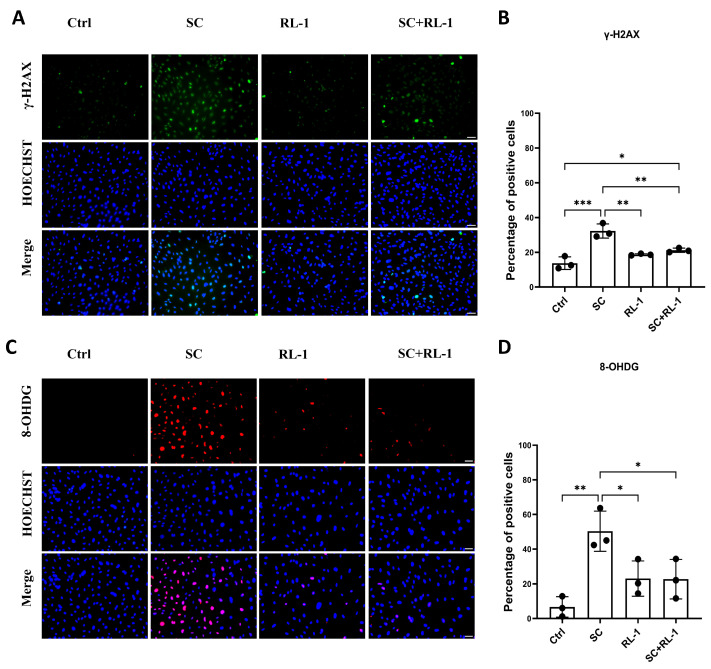
(**A**) Representative immunofluorescence images showing γ-H2AX (green), nuclei (Hoechst, blue) and merged images in HUVECs after 2 h treatment under the indicated conditions (Ctrl, SC, RL-1, and SC + RL-1). (**B**) Quantification of γ-H2AX staining. (**C**) Representative immunofluorescence images showing 8-OHDG (red), nuclei (Hoechst, blue) and merged images in HUVECs after 2 h treatment under the indicated conditions. (**D**) Quantification of 8-OHDG staining. Scale bar = 50 μm. Data are presented as mean ± SD (*n* = 3). Statistical significance was determined using one-way ANOVA with Tukey’s post hoc test. Significance is indicated as: * *p* < 0.05, ** *p* < 0.01, *** *p* < 0.001.

In line with the ROS expression data, SC provoked a clear increase in DNA damage in HUVECs, as indicated by elevated γ-H2AX positivity, which was significantly attenuated by treatment in combination with Rapalink-1 (Percentage of positive cells: Control = 13.69 ± 3.62%; SC = 32.24 ± 4.03%; Rapalink-1 = 18.66 ± 0.49%; SC + Rapalink-1 = 21.13 ± 1.21%; *p* = 0.0002; Figure 2A,B).

We next assessed oxidative DNA base lesions using 8-OHDG. SC markedly increased the proportion of 8-OHDG-positive cells, which was reversed by Rapalink-1 treatment (Percentage of positive cells: Control = 6.66 ± 5.89%; SC = 50.30 ± 11.62%; Rapalink-1 = 22.97 ± 10.12%; SC + Rapalink-1 = 22.64 ± 11.35%; *p* = 0.0047; Figure 2C,D).

The corresponding experiments in SMCs yielded comparable trends and are provided in Supplementary Figure S2.

### 2.3. Rapalink-1 Alleviates SC-Induced Premature Senescence

Oxidative stress and DNA damage are well-established drivers of cellular senescence; accordingly, we assessed SA-β-gal activity, Lamin B1 expression, and p21, and found consistent senescence-associated changes in both HUVECs and SMCs.

**Figure 3:**
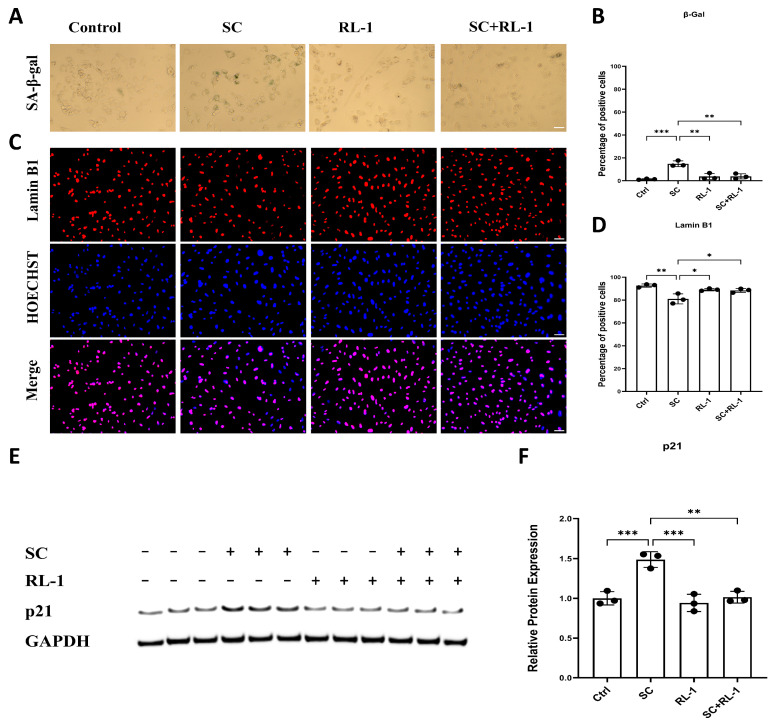
(**A**) Representative images of SA-β-gal staining in HUVECs after 24 h treatment under Control, SC, RL-1, and SC + RL-1 conditions. (**B**) Quantification of SA-β-gal-positive cells. (**C**) Representative immunofluorescence images of Lamin B1 (red), Hoechst nuclear counterstaining (blue) and merged images in HUVECs after 24 h treatment. (**D**) Quantification of Lamin B1-positive cells. (**E**) Representative immunoblotting of p21 expression after 24 h treatment. (**F**) Densitometric analysis of p21 protein levels normalized to GAPDH. Scale bar = 50 μm. Data are presented as mean ± SD (*n* = 3). Statistical significance was determined using one-way ANOVA with Tukey’s post hoc test. Significance is indicated as: * *p* < 0.05, ** *p* < 0.01, *** *p* < 0.001.

In HUVECs, SC markedly increased the proportion of SA-β-gal–positive cells, whereas Rapalink-1 alone induced only a mild change; importantly, treatment in combination with Rapalink-1 significantly suppressed SC-induced senescence (Percentage of SA-β-gal–positive cells: Control = 1.32 ± 0.47%; SC = 14.81 ± 2.53%; Rapalink-1 = 3.71 ± 2.64%; SC + Rapalink-1 = 3.81 ± 2.27%; *p* = 0.0003; Figure 3A,B).

Consistent with a senescent phenotype, SC exposure led to a pronounced reduction in Lamin B1 positivity, which was effectively preserved by treatment in combination with Rapalink-1 (Percentage of Lamin B1–positive cells: Control = 92.69 ± 1.41%; SC = 81.04 ± 4.37%; Rapalink-1 = 89.15 ± 1.04%; SC + Rapalink-1 = 88.43 ± 1.74%; *p* = 0.0029; Figure 3C,D).

To further confirm senescence-associated growth arrest, we examined p21 protein levels by Western blotting. SC significantly upregulated p21 expression, whereas Rapalink-1 attenuated this induction in the SC + Rapalink-1 group (Figure 3E,F).

In SMCs, SA-β-gal staining did not show a consistent increase following SC treatment and was therefore not used as the primary readout of senescence in this cell type. Instead, senescence-associated changes were supported by the reduction of Lamin B1 and the induction of p21, both of which were attenuated by Rapalink-1 co-treatment (Supplementary Figure S3).

### 2.4. Rapalink-1 Attenuates SC-Induced SASP-Associated Inflammatory and ECM Remodeling Responses

To further evaluate the effects of Rapalink-1 on the senescence-associated secretory phenotype (SASP) and extracellular matrix (ECM) remodeling, we assessed transcriptional changes in key inflammatory and matrix-related genes in HUVECs and SMCs.

**Figure 4:**
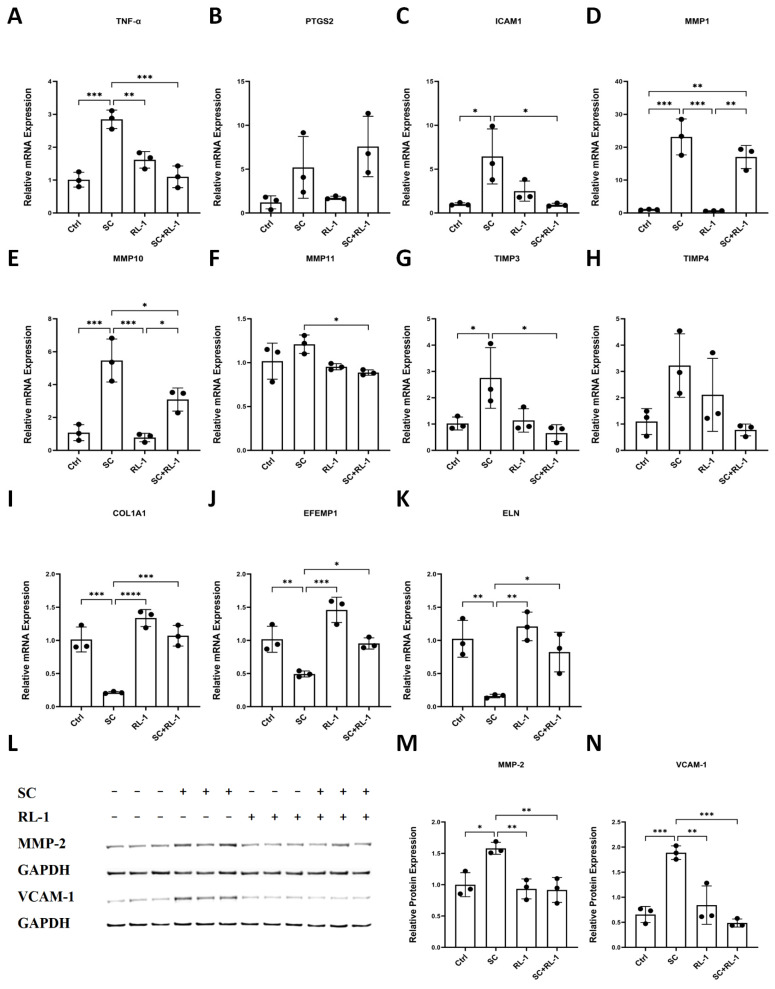
(**A**–**H**) Relative mRNA expression of TNF-α, PTGS2, ICAM1, MMP1, MMP10, MMP11, TIMP3, and TIMP4 in HUVECs after 24 h treatment. (**I**–**K**) Relative mRNA expression of selected ECM-associated genes (Collagen1, Fibulin3, and ELN) in SMCs after 24 h treatment. (**L**) Representative immunoblots of MMP-2 and VCAM-1 in HUVECs after 24 h treatment, with GAPDH as the loading control. (**M**,**N**) Quantification of MMP-2 and VCAM-1 protein expression normalized to GAPDH in HUVECs. Data are presented as mean ± SD (*n* = 3). Statistical significance was determined using one-way ANOVA with Tukey’s post hoc test. Significance is indicated as: * *p* < 0.05, ** *p* < 0.01, *** *p* < 0.001, **** *p* < 0.0001.

Analysis of the qPCR data showed that Rapalink-1 attenuated several SC-induced transcriptional changes associated with inflammatory/SASP- and matrix-remodeling responses. Representative HUVEC data are shown in Figure 4A–H, in which Rapalink-1 reduced the SC-induced upregulation of TNF-α, ICAM1, MMP1, MMP10, and TIMP3, whereas PTGS2 remained elevated despite Rapalink-1 treatment. MMP11 showed only modest changes, and TIMP4 showed a downward trend in the SC + RL-1 group. Given the prominent ECM-related changes in SMCs, selected SMC ECM-associated transcripts (COL1A1, EFEMP1, and ELN) are shown in Figure 4I–K. The full qPCR results for HUVECs and SMCs are provided in Table 1 and Table 2, and an overview heatmap comparing SC + Rapalink-1 versus SC is presented in Supplementary Figure S4. Notably, the magnitude and direction of regulation differed between HUVECs and SMCs, indicating cell-type-specific responses.

To provide representative protein-level support for selected SASP/ECM-associated alterations, we performed Western blot analysis of MMP-2 and VCAM-1 in both HUVECs and SMCs. Additional qPCR data for selected markers, including MMP2 and VCAM1, are provided in Supplementary Table S3. SC significantly increased the protein expression of MMP-2 and VCAM-1, whereas treatment in combination with Rapalink-1 reduced their expression toward control levels. Representative data from HUVECs are shown in the main figures, while corresponding analyses in SMCs are provided in the Supplementary Information (Supplementary Figure S4).

### 2.5. Rapalink-1 Modulates NF-κB- and MAPK-Related Signaling and Alters Downstream mTOR-Related Readouts

Oxidative stress is a major trigger of inflammatory signaling in vascular cells. We therefore assessed NF-κB- and MAPK-related signaling by measuring total and phosphorylated p65, p38, and ERK1/2 in HUVECs and SMCs under the indicated conditions.

**Figure 5:**
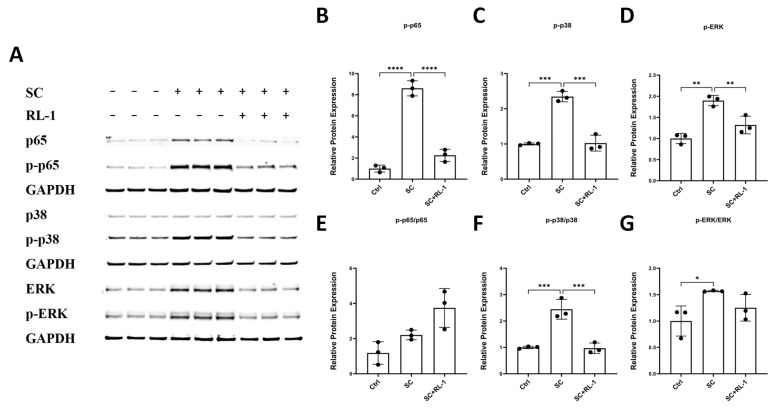
(**A**) Representative blots of the indicated proteins in HUVECs after 24 h treatment. (**B**–**G**) Quantification of the relative protein expression of p-p65, p-p38, p-ERK, p-p65/p65, p-p38/p38 and p-ERK/ERK in HUVECs. Protein expression was normalized to GAPDH. Data are presented as mean ± SD (*n* = 3). Statistical significance was determined using one-way ANOVA with Tukey’s post hoc test. Significance is indicated as: * *p* < 0.05, ** *p* < 0.01, *** *p* < 0.001, **** *p* < 0.0001.

In HUVECs, SC was associated with increased abundance of p-p65, p-p38, and p-ERK, and these phospho-signals were reduced in the presence of Rapalink-1 (Figure 5A–D). The attenuation was most evident for p-p65 and p-p38, whereas p-ERK also showed a decrease in the SC + RL-1 group. As phosphorylation-dependent changes are commonly used as readouts of NF-κB- and MAPK-related signaling, phosphorylated proteins were used as the principal readouts, while the corresponding total proteins were also assessed to support interpretation.

Among the phospho/total ratios, p-p38/p38 showed a pattern broadly consistent with the phospho-protein abundance data, whereas the corresponding ratios for p65 and ERK were less clearly aligned with the changes observed in phospho-protein abundance alone (Figure 5E–G), suggesting that SC and Rapalink-1 may influence both phosphorylation state and total protein abundance in a target-dependent manner. Overall, these findings support modulation of NF-κB- and MAPK-related signaling by Rapalink-1 in HUVECs. Comparable trends were also observed in SMCs and are presented in the Supplementary Information (Supplementary Figure S5).

**Figure 6:**
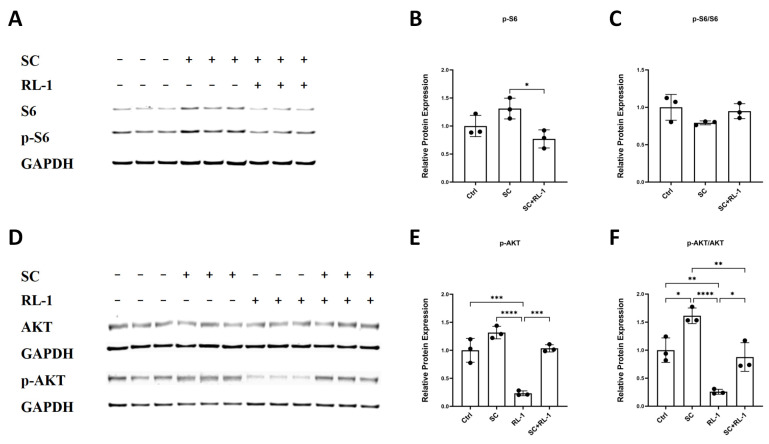
(**A**) Representative immunoblot images showing total S6, phospho-S6 (p-S6) and GAPDH in HUVECs after 24 h treatment. (**B**,**C**) Quantification of relative protein expression levels of p-S6, and the p-S6/S6 ratio. (**D**) Representative immunoblot images for AKT and p-AKT in HUVECs after 24 h treatment. (**E**,**F**) Quantification of p-AKT and p-AKT/AKT ratio. Protein expression was normalized to GAPDH. Data are presented as mean ± SD (*n* = 3). Statistical significance was determined using one-way ANOVA with Tukey’s post hoc test. Significance is indicated as: * *p* < 0.05, ** *p* < 0.01, *** *p* < 0.001, **** *p* < 0.0001.

Because Rapalink-1 is a dual mTORC1/2 inhibitor, we next examined pathway activity to confirm on-target signaling modulation in HUVECs and SMCs. Notably, neither total mTOR nor phosphorylated mTOR levels showed significant differences among groups, and the p-mTOR/mTOR ratio remained unchanged (Supplementary Figure S6), suggesting that SC does not measurably alter mTOR phosphorylation status at this time point. We therefore focused on downstream pathway outputs. Immunoblotting revealed that SC activated S6 signaling, whereas Rapalink-1 attenuated this response. Specifically, p-S6 levels were elevated following SC exposure and decreased upon Rapalink-1 treatment (Figure 6A,B). Notably, the p-S6/S6 ratio did not show a significant difference among groups, suggesting that the overall increase in p-S6 was largely accompanied by changes in total S6 abundance rather than a marked shift in phosphorylation stoichiometry (Figure 6C). These findings suggest that SC may regulate mTOR pathway output primarily at the level of downstream effectors rather than through detectable changes in mTOR phosphorylation.

AKT-related signaling was assessed by measuring p-AKT and the p-AKT/AKT ratio. SC increased p-AKT levels in HUVECs, whereas Rapalink-1 alone markedly reduced p-AKT and SC + RL-1 partially attenuated the SC-associated increase (Figure 6D,E). SC also significantly increased the p-AKT/AKT ratio in HUVECs, while Rapalink-1 alone markedly suppressed this ratio. Importantly, co-treatment with Rapalink-1 partially reversed SC-induced AKT activation, restoring p-AKT/AKT toward baseline levels (Figure 6D,F). SMCs exhibited a comparable pattern of S6 and AKT pathway modulation; however, these data are presented in the Supplementary Figures (Supplementary Figure S5).

Collectively, these results indicate that SC enhances downstream mTOR-related signaling in both HUVECs and SMCs, whereas Rapalink-1 attenuates this pathway activation despite minimal detectable changes in mTOR phosphorylation itself.

## 3. Discussion

Cigarette smoking accelerates vascular aging and degeneration through complex, interdependent mechanisms involving oxidative stress, inflammation, and ECM remodeling [1,2,3,4,32]. Although each of these processes has been linked to smoking-related vascular pathology, how they converge at the cellular signaling level remains incompletely defined [32,33]. Here, we show that SC induces a coordinated stress-associated phenotype in vascular cells and dual mTORC1/2 inhibition with Rapalink-1 attenuates several linked components of this response.

A central observation of this study is that oxidative stress is closely linked to downstream genotoxic injury and senescence establishment following smoke exposure. SC induced a rapid increase in intracellular ROS in HUVECs [21,22,23,34]. This oxidative burst was accompanied by a broader reprogramming of redox-responsive genes, including induction of oxidant-generating and antioxidant defense pathways [5,6,7,12]. Comparable oxidative responses were also observed in SMCs, supporting the view that smoke-induced redox stress is not restricted to endothelial cells.

Consistent with an oxidative-to-genotoxic progression, SC exposure was associated with accumulation of canonical DNA damage markers in HUVECs, including increased γ-H2AX positivity and elevated 8-OHDG, indicative of DNA damage signaling and oxidative DNA base lesions, respectively [12,14,15,16,24]. Similar trends in SMCs further support the generalizability of this oxidative–genotoxic axis. Mechanistically, oxidative and genotoxic stress are known to reinforce one another: persistent ROS can induce DNA lesions and mitochondrial dysfunction, while unresolved DNA damage activates the DNA damage response (DDR) and checkpoint pathways that promote stable growth arrest [14,15,16,33,35]. When stress is sustained, this state can transition into irreversible senescence, which is further stabilized by nuclear and chromatin remodeling, including depletion of Lamin B1 and reinforcement of inflammatory transcriptional programs that underpin SASP outputs [36]. In this framework, SC-induced increases in SA-β-gal activity, Lamin B1 loss, and induction of p21 protein are consistent with DDR-associated senescent growth arrest in our model.

Notably, Rapalink-1 attenuated this upstream oxidative–genotoxic cascade at multiple levels. Co-treatment significantly reduced SC-induced ROS accumulation. This effect was accompanied by a partial restoration of cellular viability under smoke stress and blunting of the maladaptive redox-responsive transcriptional activation pattern, suggesting that mTOR-centered signaling may influence oxidant burden and/or redox compensation under smoke stress [25,26,27,28,31,37]. An exception was HMOX1 in HUVECs, which remained elevated despite Rapalink-1 co-treatment, suggesting that the effects of Rapalink-1 are selective rather than uniformly suppressive, possibly preserving aspects of adaptive cytoprotective signaling while dampening maladaptive stress amplification. In parallel, Rapalink-1 decreased γ-H2AX and 8-OHDG signals, indicating reduced downstream DNA damage. At the phenotypic level, Rapalink-1 suppressed senescence-associated features, including reduced SA-β-gal positivity, preservation of Lamin B1, and diminished induction of p21 protein. Together, these findings are consistent with the interpretation that dual mTORC1/2 inhibition can decouple smoke-induced oxidative stress from subsequent genotoxic injury and senescence stabilization in vascular cells challenged by tobacco-derived toxins.

A major pathological consequence of vascular senescence is the emergence of SASP-associated programs that can amplify chronic inflammation and promote ECM remodeling [14,15,17,35]. In this context, senescence and SASP are mechanistically coupled: persistent stress signaling that establishes senescence also supports sustained inflammatory transcriptional output that reinforces and propagates tissue dysfunction. Consistent with this model, SC induced broad upregulation of SASP-associated transcripts in vascular cells [17,18,38,39]. Importantly, these transcriptional changes were accompanied by signatures consistent with adverse tissue remodeling: SC reduced expression of key structural ECM genes such as ELN, collagen1, and fibulin3, whereas Rapalink-1 partially restored these transcripts toward control levels [18,39,40]. Rapalink-1 also reduced SC-induced expression of representative inflammatory/ECM-associated effectors at the protein level (e.g., MMP-2 and VCAM-1). Collectively, these data suggest that Rapalink-1 not only attenuates senescence-associated cellular phenotypes but also modulates downstream SASP-associated inflammatory and ECM-related molecular changes induced by SC. This raises the possibility that the effect of Rapalink-1 is not limited to reducing cellular injury, but may also involve reshaping the secretory and inflammatory phenotype of stressed vascular cells, in line with the recognized role of mTOR in SASP regulation.

Mechanistically, our data implicate coordinated suppression of NF-κB and MAPK signaling as a key node through which Rapalink-1 dampens smoke-induced inflammatory transcription and SASP-associated outputs. NF-κB and MAPK pathways are well-established drivers of inflammatory gene expression and are strongly implicated in maintaining SASP-related programs [41,42,43,44,45].

In our study, SC was associated with increased abundance of phosphorylated p65 (NF-κB), p38, and ERK (MAPK), whereas Rapalink-1 reduced these phosphorylation-related readouts in HUVECs. Comparable pathway modulation was observed in SMCs, supporting a conserved mechanism across vascular cell types. These signaling effects provide a plausible mechanistic basis for the broad suppression of SASP-associated transcripts and the partial preservation of ECM-related gene expression, and suggest that dual mTORC1/2 inhibition disrupts a self-reinforcing circuit linking stress signaling, inflammatory transcription, and senescence/SASP maintenance. The relationship between cigarette smoke exposure and mTOR signaling is likely context-dependent rather than uniformly activating. In this regard, previous work in pulmonary injury models identified RTP801/REDD1, a suppressor of mTOR signaling, as an essential mediator of cigarette smoke-induced injury, suggesting that smoke exposure can also engage mTOR-inhibitory stress programs in a tissue- and model-specific manner [46]. Against this background, the present findings are best interpreted as evidence for modulation of selected downstream mTOR-related signaling readouts in vascular cells under the current experimental conditions rather than a universal pattern of canonical mTOR activation by smoke.

An additional aspect of our findings is the modulation of AKT signaling by Rapalink-1 under smoke stress. SC exposure increased the p-AKT/AKT ratio, consistent with engagement of adaptive pro-survival signaling in response to sustained cellular stress, whereas Rapalink-1 alone reduced basal AKT activity; notably, co-treatment partially normalized stress-induced AKT activation toward baseline levels [25,26,47,48,49]. In parallel, Rapalink-1 reduced SC-associated p-S6 abundance, supporting on-target modulation of downstream mTOR-related signaling. This bidirectional tuning of AKT output may be relevant in senescence biology, where excessive survival signaling can stabilize senescent cells and reinforce SASP programs, whereas overly aggressive suppression could compromise viability. Thus, dual mTORC1/2 inhibition may recalibrate adaptive AKT signaling while restraining pathological inflammatory and metabolic pathway activation.

Taken together with previous studies, our findings support the view that vascular senescence is one of the mechanisms linking cigarette smoke exposure to vascular injury and degeneration [12,13,14,15,16,32,33,35,50]. Rather than causing only acute cellular damage, cigarette smoke may impose sustained oxidative and inflammatory stress on the vessel wall, thereby promoting endothelial dysfunction and genotoxic injury [5,6,7,8,34,50,51,52]. When such stress persists, vascular cells may acquire a senescence-associated phenotype characterized by growth arrest and phenotypic remodeling [14,15,16,33,36]. In turn, these cells may develop a SASP-associated inflammatory profile together with ECM-remodeling changes, which can further disturb vascular homeostasis and amplify local tissue injury [14,15,17,18,38,39].

In this context, oxidative stress, DNA damage, cellular senescence, and maladaptive matrix remodeling are better viewed as interconnected processes rather than isolated events [32,33,35,38,39,50]. Their interaction is likely to contribute to vascular aging and may be relevant to smoking-related vascular diseases, including atherosclerotic change and aneurysm-associated wall degeneration [3,4,9,10,11,32,39,52]. Our study may provide mechanistic support for the idea that targeting senescence-associated signaling pathways may help limit smoke-induced vascular injury and may be relevant to strategies aimed at preserving vascular health during aging [20,30,31].

Several limitations should be acknowledged. This study was performed in vitro using cultured endothelial and smooth muscle cells and therefore does not fully recapitulate the biomechanical, immune, and hemodynamic complexity of the vascular wall in vivo. Future studies employing animal models of chronic smoke exposure and vascular disease will be needed to validate these findings and assess the long-term safety and efficacy of dual mTORC1/2 inhibition.

## 4. Materials and Methods

### 4.1. Cell Culture

The HUVEC models were procured from PromoCell (Heidelberg, Germany). Endothelial cell medium (C-22210, PromoCell, Heidelberg, Germany) containing endothelial growth factors (C-39215, PromoCell, Heidelberg, Germany) was used to maintain the HUVECs at 37 °C in a humidified environment with 5% CO_2_. The SMC models were procured from PromoCell (Heidelberg, Germany). Smooth muscle cell medium (C-22262) supplemented with smooth muscle growth factors (C-39267) was used to maintain SMCs at 37 °C in 5% CO_2_. Upon arrival, the cells were thawed and seeded in T75 culture flasks. The cells were passaged when they reached 80–90% confluence. For passaging, the cells were washed with PBS and incubated with trypsin for 4 min at 37 °C and 5% CO_2_. The cells were seeded at a density of 5000 cells/cm^2^ in new cell culture plates. All experiments with HUVECs and SMCs were performed at passage 7. All experiments were performed with three biological replicates.

### 4.2. Tobacco Smoke Condensate Preparation

Tobacco smoke condensate (SC) was prepared by smoking commercial cigarettes on an automated smoking machine following standard procedures for mainstream smoke collection. The smoke was passed through a Cambridge filter pad to collect the particulate phase. The pad was extracted with absolute ethanol, and the solvent was evaporated under a gentle nitrogen stream at room temperature. The dried residue was weighed and dissolved in DMSO to prepare a 100 mg/mL stock solution. For cell exposure, the stock was diluted in culture medium to the desired concentrations (final DMSO ≤ 0.1% *v*/*v*).

### 4.3. MTT Assay

Cell viability was evaluated using the MTT (3-(4,5-dimethylthiazol-2-yl)-2,5-diphenyltetrazolium bromide) assay. HUVECs and SMCs were seeded in 96-well plates at a density of 5000 cells/cm^2^ and incubated overnight at 37 °C in a humidified atmosphere with 5% CO_2_ to allow for cell attachment. The next day, the culture medium was replaced with fresh medium containing tobacco smoke condensate (50 µg/mL), 200 pM Rapalink-1, or a combination of tobacco smoke condensate and Rapalink-1. The concentrations used in the present study (SC: 50 µg/mL; Rapalink-1: 200 pM) were chosen based on previous studies and empirical observations under our experimental conditions. Cells cultured in drug-free medium served as the control group. After 24 and 48 h of treatment, 10 µL of MTT solution (5 mg/mL in PBS) was added to each well, and cells were incubated for an additional 3–4 h at 37 °C. Following incubation, the culture medium was carefully removed, and 100–150 µL of DMSO was added to dissolve the formazan crystals. Cell viability was assessed by measuring the optical density (OD) at 550 nm with reference at 655 nm. Background values were subtracted. All experiments were performed in triplicate.

### 4.4. DCFH-DA Staining

To investigate the accumulation of cellular ROS, 5000 cells/cm^2^ HUVECs and SMCs were seeded in a 96-well plate. The next day, the medium was replaced with fresh medium either containing tobacco smoke condensate (50 µg/mL), 200 pM Rapalink-1 or tobacco smoke condensate combined with Rapalink-1. HUVECs and SMCs medium alone was used as a control. After 2 h of treatment, 10 µM of the fluorescence probe 2,7-dichlorofluorescein diacetate (DCFH-DA, D6883, Sigma-Aldrich, St. Louis, MO, USA) was added to the cells. The cells were incubated with DCFH-DA at 37 °C for 30 min in the dark. The cells were washed three times with a serum-free medium. After DCFH-DA incubation and washing, the nuclei were counterstained with Hoechst. Images were acquired using identical microscope settings for all groups within each experiment and analyzed using ImageJ (version 1.53c; National Institutes of Health, Bethesda, MD, USA). DCF-positive cells were quantified relative to the total number of Hoechst-positive nuclei in the same field.

### 4.5. Immunofluorescence Staining

The cells were treated with different conditions (as described in the previous section) for 2 h for 8-OHDG, γ-H2AX staining and 24 h for Lamin B1 staining. After washing cells thrice with PBS, the cells were fixed with 4% paraformaldehyde for 10 min at RT. After washing cells thrice with PBS, for permeabilization, the cells were treated with 0.2% Triton™ X-100 at RT for 10 min. For blocking, the cells were incubated with 5% bovine serum albumin (BSA) at RT for 1 h. The cells were incubated overnight with primary antibodies 8-OHDG, γ-H2AX and Lamin B1 (Supplementary Table S1) at 4 °C. The next day, after washing cells thrice with PBS, the cells were incubated with secondary antibodies (Supplementary Table S1) for 1 h at RT. Hoechst (Sigma-Aldrich) was used for nuclear staining. The images were captured using a Leica DMi8 Inverted Microscope and the compatible LAS-X Life Science Microscope Software, version 3.7.5.24914 (Leica Application Suite X) Platform. All immunofluorescence experiments were independently repeated three times, with triplicate wells per condition in each experiment. One predefined central field was captured from each well using identical imaging settings within each experiment. Images were processed in Fiji/ImageJ (version 1.53c) (National Institutes of Health, Bethesda, MD, USA) using identical brightness/contrast settings. Positive cells were manually counted in a masked manner and expressed as a percentage of the total number of Hoechst-positive nuclei in the same field.

### 4.6. Western Blot

For protein analysis, cells were treated with tobacco smoke condensate (50 µg/mL) or tobacco smoke condensate combined with Rapalink-1 for 24 h. Untreated HUVECs and SMCs were used as controls. RIPA buffer was used for total protein extraction. DC Protein Assay Kit (500-0116, Bio-Rad, Hercules, CA, USA) was used to quantify protein concentration. Subsequently, 30 µg of total protein under reducing conditions was loaded onto a 12% sodium dodecyl sulfate-polyacrylamide gel. For the first 10 min, electrophoresis was conducted at 60 Volts, followed by 90 Volts for 60–90 min. The separated proteins were then transferred onto a 0.45 μm pore-size nitrocellulose membrane at 250 mA for 120 min. The membranes were blocked for one hour with a 5% bovine serum albumin (BSA) solution in 0.05% TBST to minimize nonspecific binding. After that, the membranes were incubated with primary antibodies (see Supplementary Table S1) in 5% BSA overnight at 4 °C on a shaking platform. Afterward, the membranes underwent 3 × 10 min washes with TBST and were subsequently exposed to secondary antibodies diluted in 0.05% TBST (refer to Supplementary Table S1) for one hour at room temperature. Densitometric analysis was performed using NIH ImageJ. For protein-abundance plots, target protein signals were normalized to GAPDH. Where indicated, phospho/total ratios were calculated separately from the corresponding phospho- and total-protein signals. Depending on the specific experiment, Western blot analyses were performed using either four treatment groups (Control, SC, Rapalink-1, and SC + Rapalink-1) or three groups (Control, SC, and SC + Rapalink-1).

### 4.7. Quantitative Polymerase Chain Reaction (qPCR)

For qPCR analysis, cells were treated with tobacco smoke condensate (50 µg/mL), 200 pM Rapalink-1, or tobacco smoke condensate combined with Rapalink-1 for 24 h. Total RNA was extracted using the Nucleo Spin RNA kit (740955.50, MACHEREY-NAGEL, Düren, Germany) according to the manufacturer’s instructions. A total of 1.2 µg of RNA was utilized for reverse transcription, accomplished using the MMLV Reverse Transcriptase kit (M1701, Promega, Walldorf, Germany), Random Hexamer Primers (48190011, Thermo Fisher Scientific, Waltham, MA, USA), and RiboLock RNase Inhibitor (EO0384, Thermo Fisher Scientific Baltics UAB, Vilnius, Lithuania). The qPCR was run using total cDNA combined with AceQ SYBR qPCR Master Mix (Q111-03, Vazyme, Nanjing, China) and primers (Supplementary Table S2) on a QuantStudio™ 3 Real-Time PCR Instrument (Applied Biosystems, Thermo Fisher Scientific, Waltham, MA, USA). The thermal cycling program consisted of an initial denaturation step at 95 °C for 8 min, followed by 40 cycles of 95 °C for 15 s, 58.9 °C for 30 s, and 72 °C for 30 s, concluding with a melting curve analysis. The relative mRNA expressions were calculated by the 2^−ΔΔCt^ method using β-actin as the reference.

### 4.8. Senescence-Associated β-Galactosidase Staining

For senescence-associated beta-galactosidase (β-Gal) staining, cells were treated for 24 h as described in the previous sections. SA β-Gal staining was performed using the Senescence Cells Histochemical Staining Kit (GALS, Sigma, St. Louis, MO, USA) following the manufacturer’s instructions. The cells were incubated with SA-β-galactosidase staining solution at 37 °C for seven hours. The staining solution was aspirated and the cells were overlaid with 70% glycerol in PBS. After staining, the cells were stored at 4 °C. The images were captured using a Leica DMi8 Inverted Microscope and the compatible LAS-X Life Science Microscope Software version 3.7.5.24914 (Leica Application Suite X) Platform. SA-β-gal staining experiments were independently repeated three times, with triplicate wells per condition in each experiment. One predefined central field was captured from each well using identical imaging settings. Positive cells were manually counted in a masked manner relative to the total number of cells in the same field.

### 4.9. Statistical Analysis

Statistical analyses were performed using GraphPad Prism (version 10.1.2). Comparisons among groups were conducted using one-way ANOVA followed by Tukey’s post hoc test. For experiments repeated independently three times, data from triplicate wells within each experiment were averaged before statistical analysis.

## 5. Conclusions

Our study demonstrates that Rapalink-1 attenuates SC-induced oxidative stress, DNA damage, senescence-associated changes, and inflammatory/ECM-related molecular responses in vascular endothelial and smooth muscle cells in vitro. These findings support Rapalink-1 as a candidate modulator of smoke-induced vascular aging and senescence-associated remodeling, warranting further validation in disease-relevant models of vascular aging and in vivo studies.

## Figures and Tables

**Table 1:** Quantification of relative mRNA expression in HUVECs (*p* values: overall one-way ANOVA across the four groups; Tukey post hoc for pairwise comparisons).

Gene	Control	SC	Rapalink-1	SC + Rapalink-1	*p*-Value
NOX4	1.06 ± 0.39	2.80 ± 0.30	0.88 ± 0.20	2.54 ± 0.81	0.0021
NOS2	1.08 ± 0.48	15.69 ± 8.49	6.81 ± 5.73	0.70 ± 0.04	0.0231
HMOX1	1.12 ± 0.67	20.07 ± 4.40	0.85 ± 0.25	25.01 ± 6.88	0.0001
SOD2	1.05 ± 0.41	7.35 ± 3.10	3.16 ± 1.21	1.18 ± 0.29	0.0058
NFE2L2	1.07 ± 0.52	3.78 ± 1.92	1.63 ± 0.94	0.73 ± 0.21	0.0371
MMP1	1.00 ± 0.14	23.13 ± 5.46	0.65 ± 0.07	17.02 ± 3.52	<0.0001
MMP10	1.07 ± 0.49	5.46 ± 1.31	0.78 ± 0.26	3.09 ± 0.70	0.0003
MMP11	1.02 ± 0.21	1.21 ± 0.11	0.95 ± 0.04	0.89 ± 0.03	0.0464
TIMP3	1.02 ± 0.25	2.75 ± 1.15	1.14 ± 0.45	0.66 ± 0.32	0.0179
TIMP4	1.09 ± 0.49	3.22 ± 1.21	2.11 ± 1.39	0.78 ± 0.22	0.0522
TNF-α	1.01 ± 0.23	2.85 ± 0.28	1.62 ± 0.25	1.10 ± 0.33	0.0001
PTGS2	1.21 ± 0.74	5.20 ± 3.52	1.73 ± 0.19	7.58 ± 3.44	0.0423
ICAM1	1.01 ± 0.16	6.44 ± 3.13	2.49 ± 1.15	0.93 ± 0.17	0.0118

**Table 2:** Quantification of relative mRNA expression in SMCs (*p* values: overall one-way ANOVA across the four groups; Tukey post hoc for pairwise comparisons).

Gene	Control	SC	Rapalink-1	SC + Rapalink-1	*p*-Value
NOX4	1.06 ± 0.42	1.74 ± 0.28	1.28 ± 0.36	1.09 ± 0.09	0.0931
NOS2	2.32 ± 2.47	15.15 ± 2.43	5.25 ± 3.00	5.80 ± 2.27	0.0014
HMOX1	1.13 ± 0.68	14.19 ± 5.70	1.28 ± 0.24	7.37 ± 1.85	0.0021
SOD2	1.07 ± 0.45	2.90 ± 1.00	1.71 ± 0.18	1.54 ± 0.68	0.0446
NFE2L2	1.08 ± 0.46	3.91 ± 1.39	1.72 ± 0.15	1.84 ± 0.92	0.0195
MMP1	1.08 ± 0.50	35.01 ± 11.49	1.38 ± 0.14	1.31 ± 0.55	0.0002
MMP10	1.59 ± 1.80	5.42 ± 1.97	2.62 ± 0.36	1.82 ± 0.64	0.0329
MMP11	1.09 ± 0.50	2.28 ± 0.61	1.57 ± 0.17	1.67 ± 0.53	0.0938
TIMP3	1.02 ± 0.27	5.12 ± 2.03	1.93 ± 0.53	1.15 ± 0.07	0.0046
TIMP4	1.01 ± 0.16	5.47 ± 2.56	2.16 ± 0.24	1.06 ± 0.55	0.0098
TNF-α	1.03 ± 0.32	3.97 ± 1.58	1.55 ± 1.05	1.52 ± 0.19	0.0227
PTGS2	1.09 ± 0.59	4.96 ± 1.08	1.71 ± 0.35	2.05 ± 0.83	0.0012
ICAM1	1.06 ± 0.45	2.71 ± 0.67	0.95 ± 0.23	1.30 ± 0.34	0.0050
ELN	1.02 ± 0.28	0.16 ± 0.03	1.21 ± 0.22	0.82 ± 0.30	0.0026
COL1A1	1.01 ± 0.19	0.21 ± 0.02	1.34 ± 0.13	1.07 ± 0.16	<0.0001
EFEMP1	1.02 ± 0.20	0.49 ± 0.05	1.46 ± 0.20	0.95 ± 0.09	0.0003

## Data Availability

The original contributions presented in this study are included in the article/Supplementary Material. Further inquiries can be directed to the corresponding author.

## Supplementary Materials

The following supporting information can be downloaded at: https://www.mdpi.com/article/10.3390/ijms27083636/s1.

## Abbreviations

The following abbreviations are used in this manuscript:

8-OHDG	8-hydroxy-2′-deoxyguanosine
AKT	protein kinase B
BSA	bovine serum albumin
PTGS2	cyclooxygenase-2
DCFH-DA	2′,7′-dichlorofluorescein diacetate
DDR	DNA damage response
ECM	extracellular matrix
ERK	extracellular signal-regulated kinase (ERK1/2)
GAPDH	glyceraldehyde-3-phosphate dehydrogenase
HMOX1	heme oxygenase-1
HUVECs	human umbilical vein endothelial cells
ICAM-1	intercellular adhesion molecule-1
Lamin B1	Nuclear lamin B1
IF	immunofluorescence
NOS2	inducible nitric oxide synthase
MAPK	mitogen-activated protein kinase
SOD2	manganese superoxide dismutase (SOD2)
MMP(s)	matrix metalloproteinase(s)
mTOR	mechanistic target of rapamycin
MTT	3-(4,5-dimethylthiazol-2-yl)-2,5-diphenyltetrazolium bromide
NF-κB	nuclear factor kappa-light-chain-enhancer of activated B cells
NOX4	NADPH oxidase 4
NFE2L2	nuclear factor erythroid 2–related factor 2 (NFE2L2)
p21	Cyclin-dependent kinase inhibitor 1
P38	p38 mitogen-activated protein kinase (MAPK14)
p65	nuclear factor kappa B subunit p65 (RelA)
RIPA	radioimmunoprecipitation assay (buffer)
ROS	reactive oxygen species
RT	room temperature
SA-β-gal	senescence-associated β-galactosidase
SASP	senescence-associated secretory phenotype
SC	smoke condensate (tobacco smoke condensate)
SMCs	smooth muscle cells (vascular)
S6	ribosomal protein S6
TBST	Tris-buffered saline with Tween-20
TIMP(s)	tissue inhibitor(s) of metalloproteinases
TNF-α	tumor necrosis factor-alpha
γ-H2AX	phosphorylated H2A histone family member X (Ser139)
VCAM1	Vascular cell adhesion molecule 1
