# Supplementary material for: Smoke Condensate-Induced Vascular Senescence and SASP Are Attenuated by Dual mTORC1/2 Inhibition with Rapalink-1
Source: Int J Mol Sci. 2026 Apr 19;27(8):3636. doi: 10.3390/ijms27083636 (PMC13116618; doi:10.3390/ijms27083636)
Supplement: Supplementary file 1 [file ijms-27-03636-s001.zip › Supplementary figure.pdf]

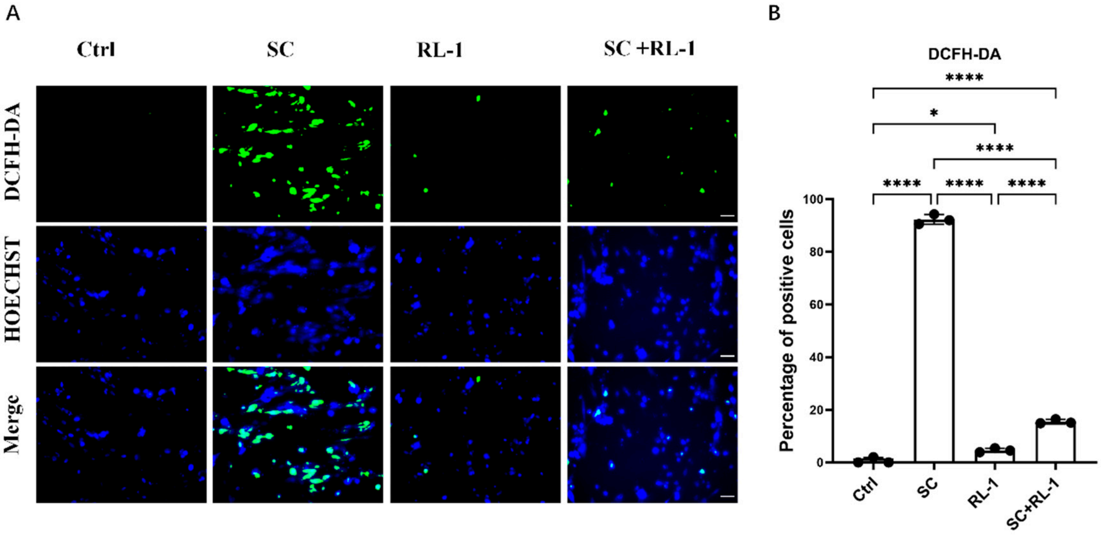

Figure S1. (A) DCFH-DA staining in SMCs. (B) Quantification of DCFH-DA–positive cells. Scale bar = 50 μm, Data are presented as mean ± SD, with individual dots representing biological replicates (n = 3). Statistical significance was determined using one-way ANOVA followed by Tukey's multiple comparisons test. Significance is indicated as: *$p < 0.05$, **$p < 0.01$, ***$p < 0.001$, ****$p < 0.0001$.

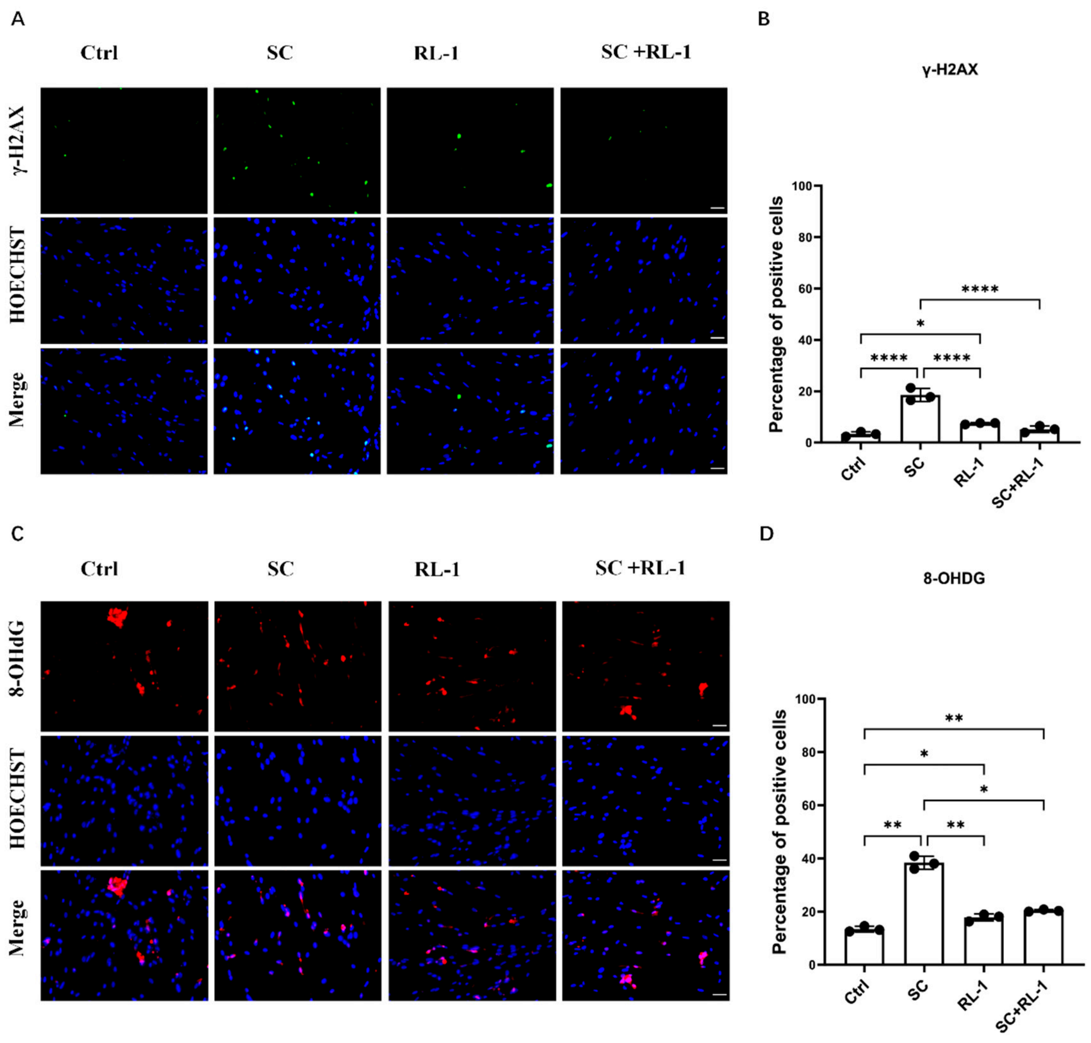

Figure S2. (A) Representative immunofluorescence images showing γ-H2AX (green) and nuclei (Hoechst, blue) in SMCs. (B) Quantification of γ-H2AX staining. (C) Representative immunofluorescence images showing 8-OHDG (red) and nuclei (Hoechst, blue) in SMCs. (D) Quantification of 8-OHDG staining. Scale bar = 50 μm. Data are presented as mean ± SD (n = 3). Statistical significance was determined using one-way ANOVA with Tukey's post hoc test. Significance is indicated as: *$p < 0.05$, **$p < 0.01$, ***$p < 0.001$, ****$p < 0.0001$.

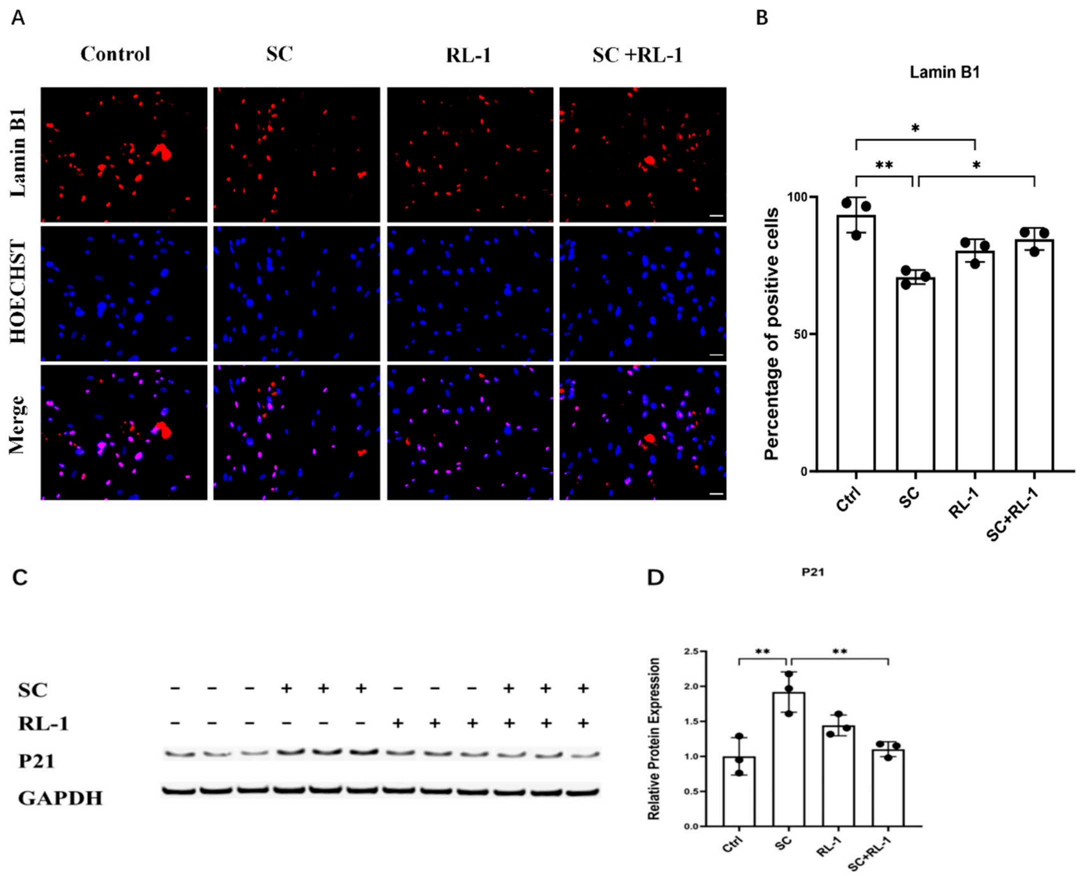

Figure S3. (A) Representative immunofluorescence images of Lamin B1 (red) with Hoechst nuclear counterstaining (blue) and merged images in SMCs. (B) Quantification of Lamin B1–positive cells. (C) Representative immunoblotting of p21 expression in SMCs. (D) Densitometric analysis of p21 protein levels normalized to GAPDH. Scale bar = 50 μm. Data are presented as mean ± SD (n = 3). Statistical significance was determined using one-way ANOVA with Tukey's post hoc test. Significance is indicated as: $^{*}p < 0.05$, $^{**}p < 0.01$, $^{***}p < 0.001$, $^{****}p < 0.0001$. Note: SA-β-gal staining was not reliably induced in SMCs under these conditions; thus, senescence was evaluated using p21, Lamin B1.

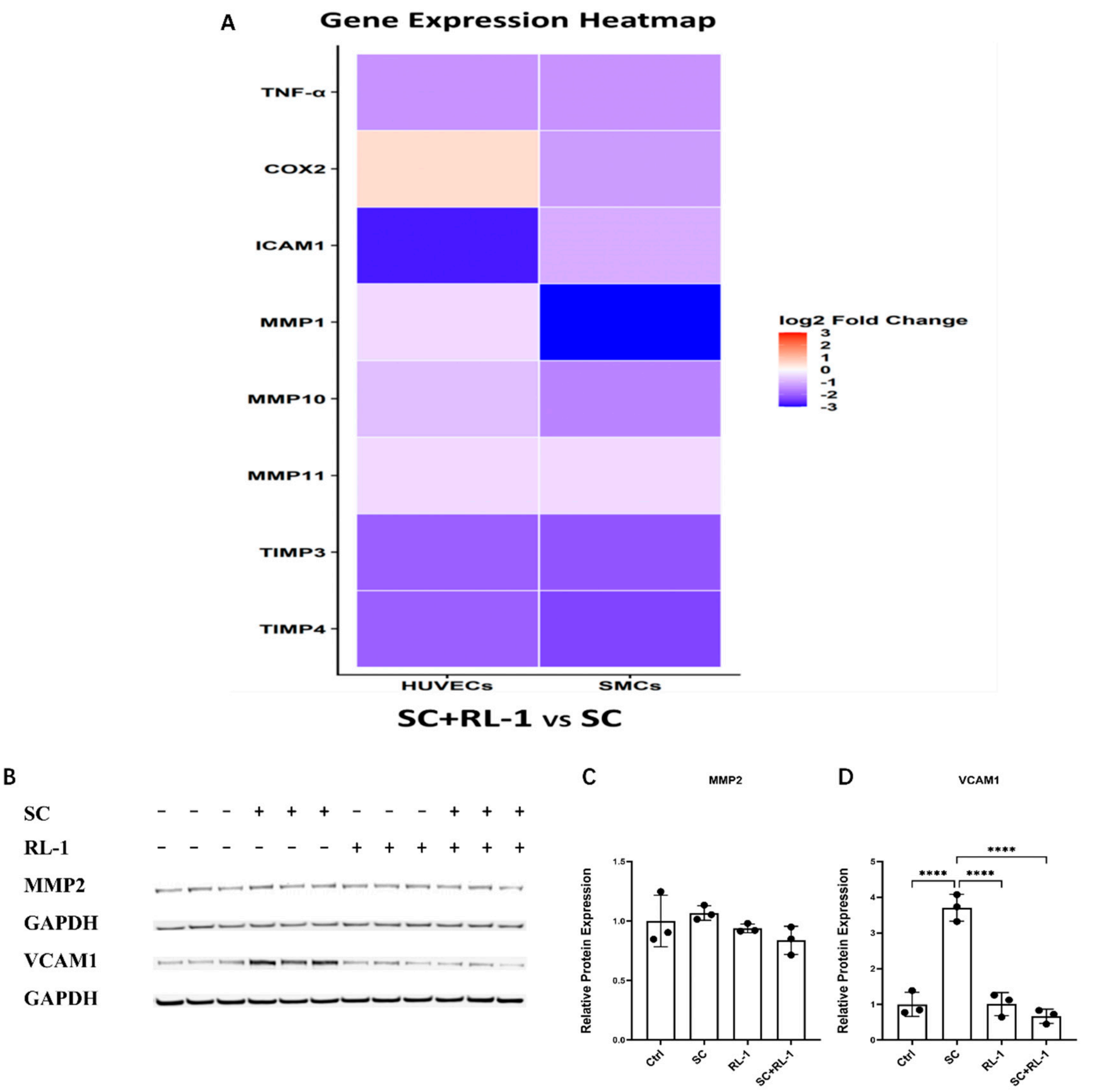

Figure S4. (A) Heatmap showing the log2 fold change of selected SASP/ECM-related transcripts in HUVECs and SMCs. (B) Representative immunoblots of MMP-2 and VCAM-1 in SMCs, with GAPDH as the loading control. (C, D) Quantification of MMP-2 and VCAM-1 protein expression normalized to GAPDH in SMCs. Data are presented as mean ± SD (n = 3). Statistical significance was determined using one-way ANOVA with Tukey's post hoc test. Significance is indicated as: $^{*}p < 0.05$, $^{**}p < 0.01$, $^{***}p < 0.001$, $^{****}p < 0.0001$.

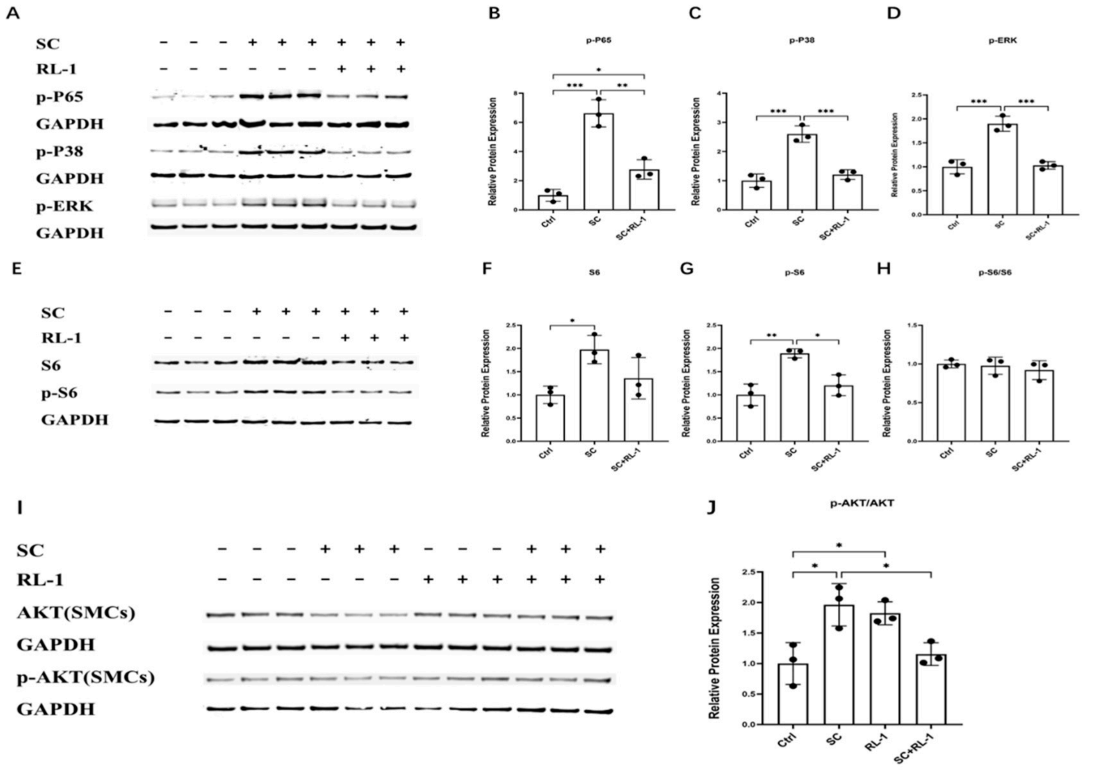

Figure S5. (A) Representative blots (p-p65, p-p38, p-ERK); (B–D) Quantification of the relative protein expression of p-p65, p-p38, and p-ERK in SMCs. (E) Representative immunoblot images showing total S6, phospho-S6 (p-S6), total AKT, phospho-AKT (p-AKT), and GAPDH in SMCs. (F–H) Quantification of relative protein expression levels of S6 (F), p-S6 (G), and the p-S6/S6 ratio (H). (I) Representative immunoblot images for AKT and p-AKT in SMCs. (J) Quantification of the p-AKT/AKT ratio. Protein expression was normalized to GAPDH. Data are presented as mean ± SD (n = 3). Statistical significance was determined using one-way ANOVA with Tukey's post hoc test. Significance is indicated as: *$p < 0.05$, **$p < 0.01$, ***$p < 0.001$, ****$p < 0.0001$.

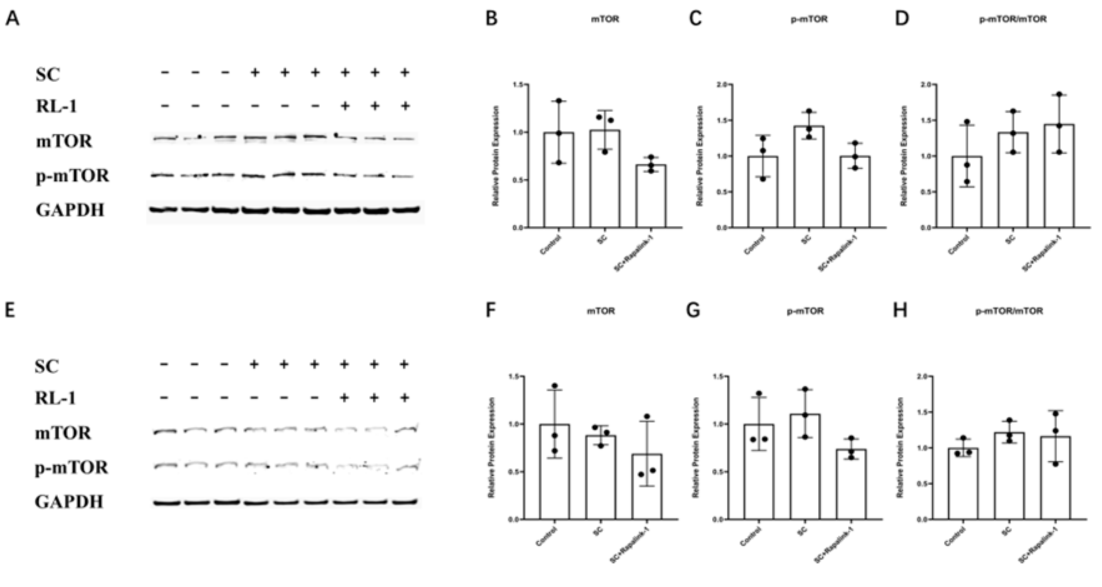

Figure S6. (A) representative blots (mTOR, p-mTOR); (B–D) quantification of the relative protein expression of mTOR, p-mTOR, and p-mTOR/mTOR in HUVECs. (E) representative blots (mTOR, p-mTOR); (F–H) quantification of the relative protein expression of mTOR, p-mTOR, and p-mTOR/mTOR in SMCs. Protein expression was normalized to GAPDH. Data are presented as mean ± SD (n = 3). Statistical significance was determined using one-way ANOVA with Tukey's post hoc test. Significance is indicated as: *$p < 0.05$, **$p < 0.01$, ***$p < 0.001$, ****$p < 0.0001$.
